# The management of heart failure in Sweden—the physician’s perspective: a survey

**DOI:** 10.3389/fcvm.2024.1385281

**Published:** 2024-05-14

**Authors:** Giulia Ferrannini, Mattia Emanuele Biber, Sam Abdi, Marcus Ståhlberg, Lars H. Lund, Gianluigi Savarese

**Affiliations:** ^1^ Division of Cardiology, Department of Medicine, Karolinska Institutet, Stockholm, Sweden; ^2^ Internal Medicine Unit, Södertälje Hospital, Södertälje, Sweden; ^3^ Department of Medical Studies, University of Trieste School of Medicine, Trieste, Italy; ^4^ Department of Internal Medicine, Acute and Reparative Medicine Theme, Karolinska University Hospital, Stockholm, Sweden; ^5^ Department of Cardiology, Heart, Vascular and Neuro Theme, Karolinska University Hospital, Stockholm, Sweden

**Keywords:** heart failure, survey, implementation, clinical inertia, guideline-directed medical therapy

## Abstract

**Aims:**

To assess the barriers to guideline-directed medical therapy (GDMT) use in heart failure (HF), diagnostic workup and general knowledge about HF among physicians in Sweden.

**Methods:**

A survey about the management of HF was sent to 828 Swedish physicians including general practitioners (GPs) and specialists during 2021–2022. Answers were reported as percentages and comparisons were made by specialty (GPs vs. specialists).

**Results:**

One hundred sixty-eight physicians participated in the survey (40% females, median age 43 years; 41% GPs and 59% specialists). Electrocardiography and New York Heart Association class evaluations are mostly performed once a year by GPs (46%) and at every outpatient visit by specialists (40%). Echocardiography is mostly requested if there is clinical deterioration (60%). One-third of participants screen for iron deficiency only if there is anemia. Major obstacles to implementation of different drug classes in HF with reduced ejection fraction are related to side effects, with no significant differences between specialties. Device implantation is deemed appropriate regardless of aetiology (69%) and patient age (86%). Specialists answered correctly to knowledge questions more often than GPs. Eighty-six percent of participants think that GDMT should be implemented as much as possible. Most participants (57%) believe that regular patient assessment in nurse-led HF clinics improve adherence to GDMT.

**Conclusion:**

Obstacles to GDMT implementation according to physicians in Sweden mainly relate to potential side effects, lack of specialist knowledge and organizational aspects. Further efforts should be placed in educational activities and structuring of nurse-led clinics.

## 1 Introduction

Heart Failure (HF) is linked with high morbidity, mortality and financial and organizational burden on the healthcare systems, with the absolute number of hospital admissions expected to increase as much as 50% in the next 25 years (1). The 2021 European guidelines on HF recommend polypharmacotherapy to reduce mortality/morbidity in HF with reduced ejection fraction (HFrEF), including renin-angiotensin-system inhibitors (RASi), angiotensin receptor-neprilysin inhibitors (ARNi), mineralocorticoid receptor antagonists (MRA), beta-blockers and sodium-glucose cotransporter 2 inhibitors (SGLT2i) (2). Device therapy, i.e., implantable cardioverter defibrillator (ICD) and cardiac resynchronization therapy (CRT), is also recommended in specific subgroups of HFrEF patients after at least three months of optimal medical therapy (2). Screening for iron deficiency is mandated for prognostic assessment, and treatment with supplementation with intravenous iron is recommended in specific subpopulations (3, 4). In the 2021 European guidelines HF with mildly reduced EF (HFmrEF) has class IIb level of evidence C recommendation for RASi, ARNi, beta-blockers and MRA, whereas in the 2023 update a class I level A recommendation for the SGLT2i empagliflozin and dapagliflozin was added, as also in HF with preserved EF (HFpEF) (2, 4).

Despite these recommendations, guideline-directed medical therapy (GDMT) is still underused in clinical practice (1, 5–10). The suggested barriers to implementation might relate to tolerability, delay in up-titration of drugs because of structural hinders, uncertainty regarding effectiveness in special patient groups (e.g., elderly, chronic kidney disease, obesity) and clinical inertia (7, 11, 12). However, large registries where studies are conducted to assess the status of and barriers to implementation rarely collect data on tolerability, side effects and explicit physicians reasoning not to treat, and therefore barriers to implementation remain poorly understood (13, 14).

Therefore, the current survey aims to investigate how Swedish physicians are aware of the current evidence behind 2021 European Society of Cardiology (ESC) guidelines recommendations on HF, how they manage HF in daily clinical practice, and what the perceived barriers to implementation of GDMT are, in order to identify specific areas of improvement.

## 2 Materials and methods

A survey comprising questions about the management of HF patients was sent by mail in two steps during fall 2021 and spring 2022 to a random sample of 10% (828) physicians selected from a database (OneKey) of 8,287 physicians, including specialists, general practitioners (GPs) and interns in Sweden, provided by IQVIA (U.S.A.), a provider of biopharmaceutical development, professional consulting and commercial outsourcing services.

The survey had a total of 42 questions divided into four different sections: (I) background of participants; (II) management of HF patients with information on workup and follow-up; (III) device therapy; (IV) pharmacological therapy and obstacles to its implementation (Supplementary Appendix 1). Missing answers were excluded from the analyses.

The characteristics of the participants and the answers were reported as percentages; comparisons were made in the overall population as well as by specialty. We stratified participants in two groups: GPs and other physicians, including specialists and residents; the latter group will be referred to as “specialists”.

## 3 Results

### 3.1 Section I: Background of participants

One-hundred sixty-eight physicians participated in the survey (response rate: 20%), 90 (54%) in the 2021 and 78 (46%) in the 2022 occasions. One-hundred and one (60%) were male and 67 (40%) were female; median age was 43 years (interquartile range—IQR: 27–77), with most participants (29%) aged 31–40, and median time after license to practice medicine 14 years (IQR: 0–51 years).

Supplementary Table 1 reports participants’ areas of specialty: 41% were GPs, 18% cardiologists and 13% internists, 24% specialists in training, and 4% had other unspecified specializations.

### 3.2 Section II. Management

Seventy-two percent of the participants reported to meet less than 10 HF patients per week, 21% meet 1–19 patients, 4% meet 20–29 patients per week, and 3% meet 30 or more HF patients per week. GPs meet significantly less HF patients compared with specialists (*p* = 0.035).

**Figure 1:**
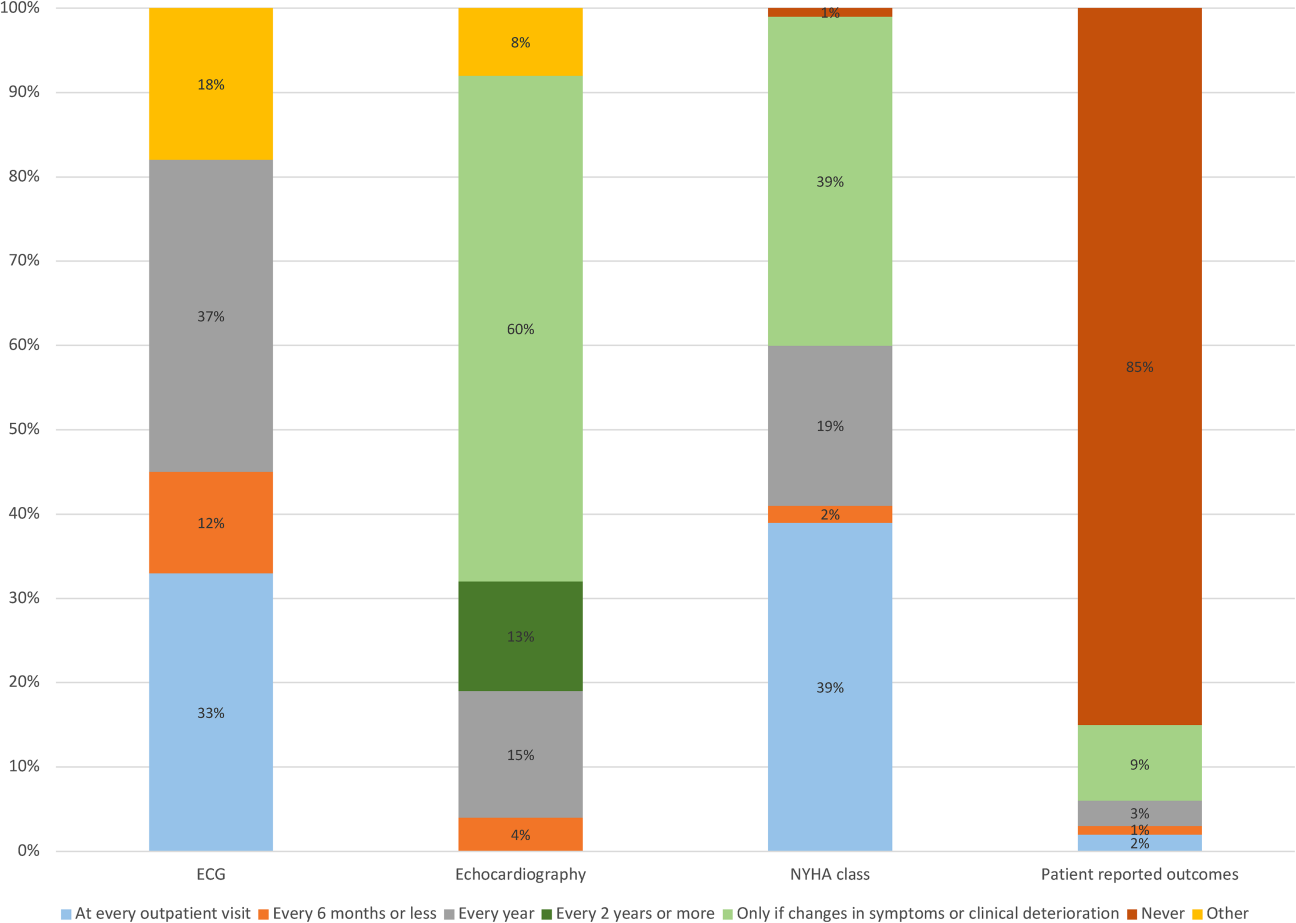
Proportions of participants answering to the question “how often do you perform an electrocardiogram/ecochardiography/NYHA class assessment/patient-reported outcome (questionnaire) assessment in your patients with heart failure”? ECG, electrocardiogram; NYHA, New York heart association.

Figure 1 displays how often participants request/perform an electrocardiogram (ECG), echocardiography, New York Heart Association (NYHA) class and patient reported outcome (questionnaire) assessments. ECG is mostly performed every year (37%, more often by GPs) or at every outpatient visit (33%, mostly by specialists), whereas echocardiography is mostly performed only if there is a change in symptoms or clinical deterioration (60%), with no difference between GPs and specialists. NYHA class assessment is performed either at every outpatient visit (39%, mostly by specialists) or if there is a clinical worsening (39%, mostly by GPs). Eighty-five percent of participants reported to never assess patient-reported outcomes, with no differences between GPs and specialists; among those who do, the most used questionnaire is the EQ-5D (7% of participants).

Most participants thought that HF patients should be followed-up in secondary/tertiary care only if there is worsening in clinical status (59%), whereas 15% believe that HF patients should be always followed-up in secondary/tertiary care, 14% believe that they should be followed-up in secondary/tertiary care only if they have HFrEF, with no differences between GPs and specialists.

Supplementary Figure 1 shows how often HF patients are scheduled for follow-up according to EF class. Most participants schedule visits once a year; however, patients with HFrEF are generally seen more often (every six months in 33% vs. 13% in HFmrEF and 4% in HFpEF, *p* < 0.001). Regarding referral to nurse-led clinics, 26% participants regularly refer their patients to nurse-led HF clinics, 26% only when up-titration and/or optimization of medical therapy is needed, 13% only when worsening of clinical status is observed, 7% never and 29% state that nurse-led HF clinics are not available at their institutions (37% of GPs and 23% of specialists, *p*-value = 0.017). Specialists tend to address their patients to nurse-led HF clinics more often than GPs do (*p* = 0.017). Most participants (57%) believe that regular patient assessment in nurse-led HF clinics might improve adherence to evidence-based medical treatment.

After starting a new HFrEF drug, 42% of participants reported that they wait for one month before re-evaluating their HF patients, 38% wait for two weeks and 4% wait for one week, with no differences between the physicians groups.

### 3.3 Section III. Device treatments

When considering implantation of ICD for primary prevention of sudden cardiac death in patients with an indication and good clinical status, most participants (69%) would recommend it regardless of aetiology and age, and 18% would not if older than 80 years. GPs tend to recommend ICD implantation with less age limitations compared to specialists (*p* < 0.0001, Supplementary Figure 2).

Most participants (86%) would recommend ICD implantation regardless of ischemia or fibrosis while 6% would recommend it only if the etiology is ischemic and 9% only if there is fibrosis at cardiac magnetic resonance. Specialists more likely recommend ICD implantation regardless of fibrosis and ischemia (87% vs. 83% of GPs, p = 0.038). After institution of optimal pharmacological therapy 72% participants wait three months before assessing for ICD/CRT, while 11% wait for one month, 15% wait for one year and 1% wait two years.

### 3.4 Section IV. Pharmacological treatments

**Figure 2:**
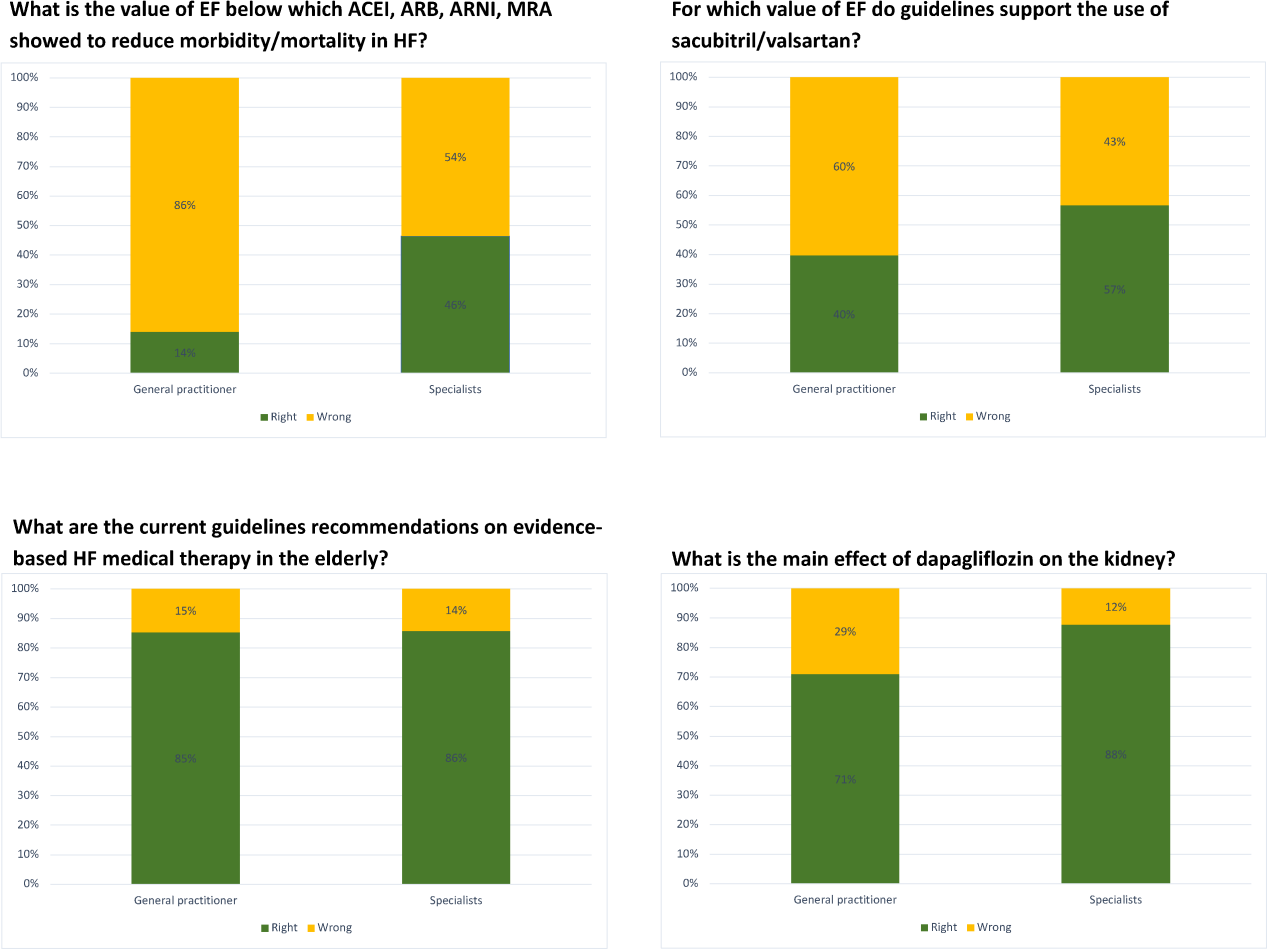
Proportions of right and wrong answers to knowledge questions about pharmacological therapy in HF, divided by specialty.

Figure 2 and Supplementary Figure 3 display the proportions of correct answers when participants were asked knowledge questions about recommended medical therapies in HF. Specialists answered significantly more correctly than GPs to questions involving EF cutoffs leading to eligibility for RASi, MRA, ARNi and SGLT2i, whereas most participants, regardless of specialty and age, think that HF therapy should be also introduced in elderly patients and are aware of the beneficial effects of dapagliflozin on the kidney. Most participants (86%) think that HF therapy should be implemented as much as possible because it reduces morbidity and mortality, and 13% think that it should be introduced to improve symptoms.

**Figure 3:**
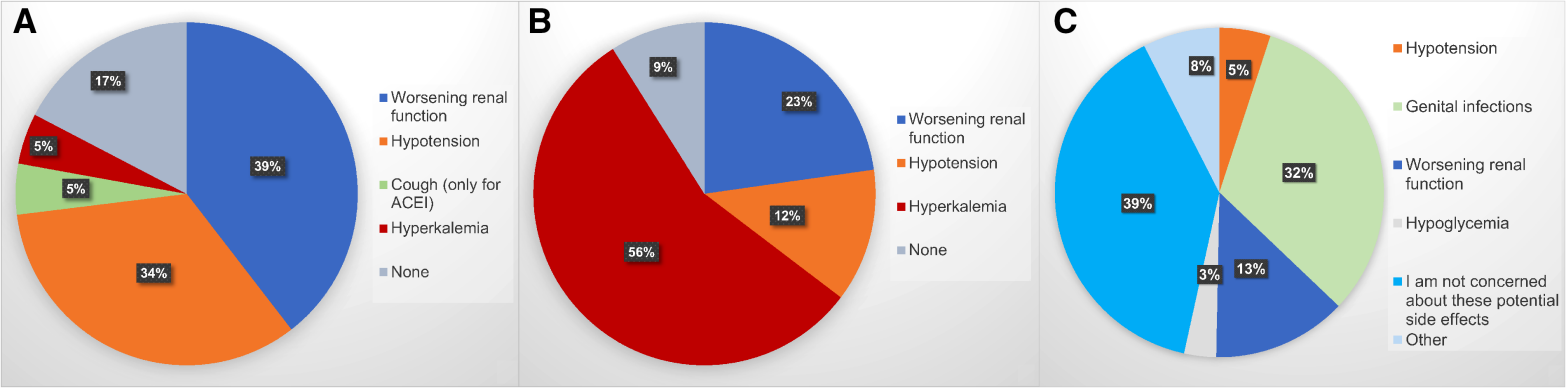
Major obstacles to implementation of guideline-directed medical therapy in heart failure with reduced ejection fraction for starting up and/or up-titrating (**A**) renin-angiotensin system blockers (including angiotensin-converting enzyme inhibitors, angiotensin receptor blockers and angiotensin receptor/neprilysin inhibitors); (**B**) mineralocorticoid receptor antagonists; (**C**) sodium-glucose co-transporter 2 inhibitors. ACEi, angiotensin-converting enzyme inhibitors.

In general, the main reason patients with HFrEF do not receive GDMT is, according to the physicians, side effects (51%), followed by the physicians not being sufficiently informed about the beneficial effects of the drugs and their indications (35%), new drugs being too expensive (8%) and patients populations differing from those in the trials (4%). Figure 3 reports major perceived obstacles in starting/up-titrating ACEi/ARB/ARNi, MRA and SGLT2i, while Supplementary Figure 4 focuses on ARNI. Worsening renal function (39%) and hypotension (34%) are the major concern as regards RASi/ARNi, hyperkalemia for MRA (56%) and genital infections for SGLT2i (32%), although most participants (39%) state that they are not concerned about side effects when using SGLT2i. For ARNi, the major obstacle is hypotension (42%). Different thresholds for blood pressure and renal function preventing the initiation and/or up-titration of medications are reported in Supplementary Figure 5, with GPs being more conservative than specialists. Thirty-two percent of participants use SGLT2i only in the presence of diabetes.

In case simulations, GPs are less likely to use at all spironolactone in a patient with potassium level of 4.7 mEq/L (26% vs. 9% of specialists, *p* = 0.013) and more likely to stop it with a potassium of 5.7 mEq/L (80% vs. 59%, *p* < 0.0001). Thirty-five percent of participants have used potassium binders, with specialists using them significantly more than GPs (43% vs. 22%, *p* = 0.004), and the provided main reason not to use them is the lack of sufficient evidence (17%).

Screening for iron deficiency is mainly performed every year (45%), and one third of participants screen for iron deficiency only in the presence of anemia.

When asked which proportion of their HF patients receives the full dose of their HF medications, 40% of participants answered 26%–50%, whereas 37% answered 50%–75%. In order to maximize adherence to guidelines, most participants (52%) think that patients should be regularly evaluated in nurse-led clinics; other strategies were follow-up in tertiary centers (28%), therapeutic implementation during hospitalization (10%) and delegating the assessment to GPs only (10%), with specialists preferring tertiary centers and GPs believing more in a primary care-centered management.

When asked in which proportion they follow a conventional sequencing approach when introducing or up-titrating drugs, 31% of participants reported that they follow it in 0%–25% of their patients, 23% in 26%–50%, 28% in 50%–75% and 18% in 76%–100% (Supplementary Figure 6). Concurrent initiation of several GDMTs at once was not assessed in this survey.

## 4 Discussion

This survey highlights important aspects to consider for improving adherence to HF guidelines in Sweden, especially as regards structure of follow-up, knowledge about GDMT and obstacles to its initiation and up-titration. In Sweden, HF patients can be followed by both GPs and specialists, and in the in-hospital setting it can also be internal medicine specialists who treat them. There are also specialized HF outpatient clinics where complex patients can be referred to. However, there are important regional differences and difficulties in allocating resources, as well as a compelling need for harmonization of healthcare processes. Therefore, surveys conducted among healthcare professionals are a valuable tool to identify specific educational needs and to develop targeted quality improvement programs, as also reported by the REWOLUTION HF program (15).

### 4.1 Follow-up and organizational aspects

Regarding the structural/organizational aspects, according to our survey ECG and echocardiography are scheduled by most physicians in accordance with guidelines (2). NYHA class assessment is performed somewhat regularly by most of the participants, and it is of importance as changes predict morbidity/mortality (16). Conversely, patient-reported outcomes are scarcely used, probably due to the fact that they are time-consuming, even though they might be a more sensitive tool to assess prognosis and they provide a more comprehensive understanding of clinical status, as compared to NYHA class (17, 18).

Regarding follow-up structure, most participants schedule visits once a year, more often for patients with HFrEF, which might be linked to the higher number of therapeutical options available in this HF phenotype, the need of GDMT up-titration and the changing clinical profile over time which might trigger dose modification (19, 20). Nurse-led HF clinics, currently mainly available in a secondary/tertiary care, are considered by most as an optimal setting for following HF patients and an excellent environment for up-titrating medications (21, 22). Indeed, in Sweden, referral to follow-up in specialty care and enrolment in the Swedish HF Registry has been reported as associated with lower risk of mortality compared with primary care (23, 24). Thus, the introduction of nurse-led clinics also in primary care in Sweden is advocated. This is in line with the fact that two thirds of participants think that HF patients should be followed-up in secondary/tertiary care whether there is worsening in clinical status, possibly indicating specialized HF care is perceived as being of greater benefit for patients (23).

### 4.2 Device therapy

Most participants would recommend primary prevention ICD implantation, if indicated, regardless of age, ischemic etiology and fibrosis, and after three months of optimal medical therapy. In a previous survey conducted among Swedish physicians in 2014, awareness of indication for ICD was 15%, therefore our results might indicate an improvement (25).

ICD implantation for primary prevention of sudden cardiac death after three months of optimized medical therapy is recommended by guidelines to reduce the risk of death, with Class IA recommendation whether the etiology is ischaemic, and Class IIa in non-ischaemic cardiomyopathy, mainly as a result of the DANISH trial not proving a statistically significant survival benefit in these patients (2, 26). However, the overall currently available evidence seems to support ICD implantation in primary prevention regardless of ischaemic aetiology and age, whereas the presence of fibrosis seems to correctly stratify patients at high risk of sudden death (27, 28). Despite this evidence, an analysis of the Swedish HF registry (SwedeHF) highlighted a wide underuse of ICD implantation in Sweden, which might be linked with HF patients often seen in primary care while decision on eventual device implantation more likely made in specialty care (9, 29).

### 4.3 Pharmacological treatment

GPs were less likely to provide correct answers to questions regarding the eligibility to treatments and case-based questions, which is reasonable to impact therapy optimization in daily clinical practice. This is understandable since GPs do not focus only on HF, but also highlights the need of tailoring educational activities on GPs beyond cardiologists and internists/geriatricians needs. Most participants were aware of renal benefits with dapagliflozin (EMPA-Kidney findings had not been released yet at the time this survey was conducted), indicating overall good dissemination of the recent studies on SGLT2i (30). Still 32% of participants would prescribe SGLT2i only in the presence of diabetes, which presumably reflects the fact that the survey was conducted right after the 2021 ESC guidelines on HF were published (2).

Most participants were aware that GDMT should be initiated regardless of age (31–33). On the other hand, data from the SwedeHF reported that patients older than 80 years remain undertreated in practice, both as regards overall use of GDMT and its up-titration (14). The discrepancy between physicianś answers to this survey and data from national registries might be linked to organizational bottlenecks preventing physicians to apply their medical knowledge, selection bias, i.e., those replying to this survey being more aware regarding specific issues related to HF care, recall bias, or only clinical inertia.

One-third of participants report that hypotension is the major concern as regards RASi/ARNi implementation. Data from the PARADIGM-HF indicate that, despite hypotension being more frequent with sacubitril/valsartan than with enalapril (2.4% vs. 1.3% discontinuations, respectively) during the run-in period, there was no difference in the rates of therapy discontinuation after randomization and patients with hypotension had similar benefits from sacubitril/valsartan (34, 35). Besides the beneficial effect of ARNi, hypotensive episodes should not discourage the initiation and up-titration of RASi/ARNi for two other reasons: (1) in patients with lower baseline systolic blood pressure (< 110 mmHg), these drugs do not induce a further reduction but an increase after four months of therapy as a result of the improvement in hemodynamics (34); (2) these drugs have flexible dosages and even non-target doses maintain a prognostic benefit across the EF spectrum (34, 36). Similar findings have been shown for MRA (37). Given the large discrepancy between the risk of hypotension linked with RASi/ARNi in clinical studies and the very high hypothetical risk of hypotension which emerges from this survey, it is reasonable to speculate that much of this risk might be perceived and not actual.

Fifty-six percent of participants deemed hyperkalemia as the major barrier to MRA use, with 16% refraining from initiation with potassium levels of 4.7 mEq/L, and 67% likely to stop it with potassium levels of 5.7 mEq/L. Overall, 35% of participants stated that they use potassium binders. European guidelines suggest for patients on MRA not to stop the treatment with a potassium level <6.5 mEq/L but to initiate potassium binders and monitoring instead, and for those not on MRA to initiate therapy with potassium-lowering agents and start with MRA once potassium is <5.0 mEq/L (2, 38). Results from the several trials have reported novel potassium binders being effective in safely maintaining normal potassium levels in patients who are on RASi/MRA (39, 40). MRA are not restarted in most patients who suspend them because of hyperkalemia and this translates into poor outcome (41). On the other hand, the high cost of novel potassium binders is perceived by physicians as an obstacle for their implementation, even though a cost-effectiveness analysis suggested that the use of MRA, ACEi and patiromer was cost-effective compared with ACEi only among patients with NYHA class III–IV (42). Importantly, there is evidence that the use of SGLT2i is associated with less hyperkalemia and discontinuation of MRA; therefore, the introduction of this class of drugs in GDMT for HFrEF will hopefully further curb this concern (43).

Worsening renal function is another major factor preventing therapy optimization, particularly as regards RASi/MRA/ARNi. Accordingly, in SwedeHF patients with chronic kidney disease were less likely to receive evidence-based treatments, even with mildly impaired renal function which is proven not to represent an impediment to the effectiveness/safety of these drugs (44–46), but results into clinical inertia preventing patients from receiving life-saving treatments.

As regards iron deficiency, only 45% of participants reported that they screen once a year and one third do it only if there is anemia. In Sweden, it is reported that less than 30% of patients with HF are actually screened and only one in five patients diagnosed with ID receive ferric carboxymaltose therapy, with anemia being an independent predictor of both screening and ferric carboxymaltose use (10, 47). A recent meta-analysis of randomized controlled trials suggests that treatment with intravenous iron reduces HF hospitalizations and cardiovascular death events (48). Therefore, more efforts should be put into educational programs to increase physicians’ knowledge about the prognostic impact of iron deficiency in HF regardless of anemia. In an environment where HF nurse-led clinics are available, intravenous iron can be administered as part of GDMT optimization.

A sequencing approach when introducing/up-titrating drugs in HFrEF was preferred to a more personalized approach by approximately half of the participants in more than half of their patients, despite the wide consensus that all four pillars of HFrEF should be prescribed simultaneously and optimized rapidly to improve HF outcomes (36, 49). The need for further implementation of simultaneous GDMT initiation in HFrEF has also been reported in a survey conducted among cardiologists in France and Switzerland (50). A good opportunity to implement this approach might be a hospitalization for HF, and indeed data from SwedeHF reported that initiation of RASi/ARNi and betablockers following a HF hospitalization is associated with lower mortality (51). As regards the timing of up-titration in HFrEF, a recent consensus from the American College of Cardiology, based on the experience of the STRONG-HF trial, states that it should be achieved by three months from a new HF diagnosis if the patient is drug-naïve, whereas the process should be faster if the patient was already on some GDMT medication (52).

### 4.4 Strengths and limitations

The main strength of this survey is that it reflects clinical practice of different professionals at different stages of their careers. The questions are constructed to explore implementation from different perspectives.

Limitations are the low response rate (20%), possibly indicating lack of time and willingness to be questioned on knowledge, and constituting a selection bias, as those who participated might be those who are more knowledgeable. Another limitation is that many participants are not heavily involved in HF care in terms of patient volume, and thus in specialty care the picture could be different. We did not inquire on possible perceived gender differences in the management of HF patients, and this should be further investigated whether physicians apply GDMT differently depending on their own and patients and physician gender (53, 54). Finally, this only refers to clinical practice in Sweden up to 2021–2022 and might not be generalizable to other settings.

## 5 Conclusions

GDMT is still underused and adherence to guidelines still limited in the real-world clinical practice. Obstacles to implementation according to physicians in Sweden mainly relate to potential side effects whose likelihood would seem overestimated in daily care, lack of specialist knowledge and organizational aspects. Further efforts should be placed in educational activities, particularly in primary care, and structuring of nurse-led clinics.

## Data Availability

The raw data supporting the conclusions of this article will be made available by the authors, without undue reservation.
