# Supplementary material for: The management of heart failure in Sweden—the physician’s perspective: a survey
Source: Front Cardiovasc Med. 2024 May 14;11:1385281. doi: 10.3389/fcvm.2024.1385281 (PMC11130511; doi:10.3389/fcvm.2024.1385281)
Supplement: Supplementary file 1 [file Datasheet1.docx]

**Supplemental material.**

**Supplemental Table 1** reports participants’ areas of specialty.

| **Type of specialty** | **n (% on the total)** |
| --- | --- |
| General practitioners | 69 (41) |
| Specialists | 99 (59) |
| Specialists in training | 41 (24) |
| Internal medicine | 21 (13) |
| Other | 6 (4) |
| Cardiologists | 31 (18) |
| Heart failure | 7 (4) |
| Electrophysiology | 4 (2) |
| Angiography | 3 (2) |
| Intensive care cardiology | 4 (2) |
| GUCH | 2 (1) |
| Pediatric cardiology | 1 (1) |
| General cardiology | 3 (2) |
| Not specified | 7 (4) |
| **Total** | **168 (100)** |

**Supplemental Figure 1**. How often participants plan a follow-up visit for their heart failure patients according to ejection fraction class. The two specialist groups differed only for HFmrEF, as general practitioners tend to visit their HFmrEF patients more often, p<0.05.

**Supplemental Figure 2**. Proportions of not recommending the implantation of an ICD for primary prevention of sudden cardiac death in a patient with an indication and a good clinical status according to age.

**Supplemental Figure 3**. Answers to knowledge questions.

What is the value of EF below which ACEI, ARB, ARNI, MRA showed to reduce morbidity/mortality in HF?

For which value of EF do guidelines support the use of sacubitril/valsartan?

What is the main effect of dapagliflozin on the kidney?

What are the current guidelines recommendations on evidence-based HF medical therapy in the elderly?

**Supplemental Figure 4**. Major obstacles to implementation of sacubitril/valsartan.

**Supplemental Figure 5**. Reasons not to initiate and/or up-titrate medications in HFrEF as regards blood pressure and renal function, according to specialty.

**Supplemental Figure 6**. Proportion of HFrEF patients in whom a conventional sequencing approach when introducing/up-titrating drugs is followed, as opposed to a more personalized approach.

**Appendix 1 – text of the survey that was sent to participants.**

**
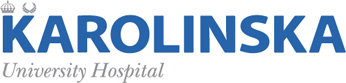

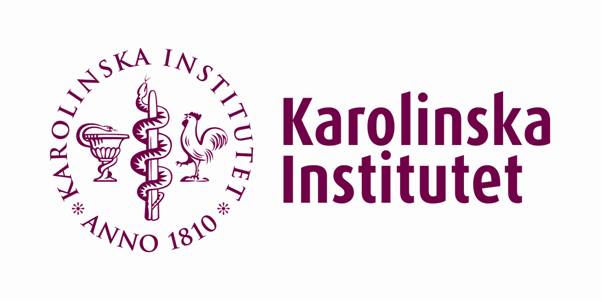
**

**Dear Colleague,**

You have been selected to participate in a scientific study regarding the management of heart failure patients in Sweden. Participation in the study is voluntary but we would very much appreciate your contribution and perspective on the treatment of heart failure.

The survey comprises 42 multiple-choice questions and takes about 5-10 minutes. Please select only answer per question.

We do not expect you to have detailed knowledge on the topic, but we kindly ask you to answer as best as you can. If you cannot answer a question, select the option *you think* is correct rather than leaving an answer blank. Please, do not search for the correct answer online or somewhere else if you are unsure about the answer, since this would make our results unreliable.

Please return the questionnaire using the postage-paid envelope included. If you prefer to answer the survey via email, please send an email to gianluigi.savarese@ki.se.

**Thank you for your participation!**

Sincerely,

Gianluigi Savarese, MD, PhD; and Lars H. Lund, MD, PhD

Division of Cardiology

Department of Medicine

Karolinska Institutet

and

Heart and Vascular Theme

Karolinska University Hospital

Stockholm, 17176 Sweden

**Contact information:**

Gianluigi Savarese, MD, PhD

E-mail: gianluigi.savarese@ki.se

**Abbreviations**

**HF:** Heart failure

**EF:** Ejection fraction

**HFrEF:** Heart failure with reduced ejection fraction

**HFpEF:** Heart failure with preserved ejection fraction

**HFmrEF:** Heart failure with mildly reduced ejection fraction

**NYHA:** New York Heart Association functional class

**ICD:** Implantable cardioverter defibrillator

**CRT:** Cardiac resynchronization therapy

**ACE:** Angiotensin converting enzyme

**eGFR:** Estimated glomerular filtration rate

**sBP:** Systolic blood pressure

**SGLT2i:** Sodium-glucose co-trasporter-2 inhibitors

**Section 1. Your background**

1. Age:

2. Sex:

Female

Male

3. Specialty:

Allmäntjänstgöring (AT) läkare

Specialiseringstjänstgöring (ST) läkare

Specialist Family medicine/General practitioner

Specialist Internal medicine

Specialist Other (If two specialties, mark the one of main focus today):

Specialist Cardiology (please specify below)

Heart failure specialist

Electrophysiology/Pacing

General cardiology

Angiography/PCI

Intensive care/Ischemia

GUCH

Other (please specify):

4. Years after license to practice medicine:

5. Years after specialist license:

**Section 2. Management of heart failure patients**

6. Approximately how many patients with HF do you see per week?

<10

10-19

20-29

30-39

40-49

≥50

7. How often do you evaluate ECG in a patient with chronic HF?

At every outpatient visit

Every sixth month or less

Once a year

Other (please specify):

8. How often do you perform / request echocardiography in your patients with HF?

Every 6 months or less

Every year

Every 2 years or more

Only if there is clinically deterioration/improvement

Other (please specify):

9. How often do you evaluate NYHA class?

At every outpatient visit

Every 6 month or less

Every year

Only if the patient reports changes in symptoms

10. Do you assess patient reported outcomes (e.g. quality of life questionnaires such as KCCQ, MLHFQ, EQ5D, etc)?

At every outpatient visit

Every 6 month or less

Every year

Only if the patient reports changes in symptoms

Never

11. Which patient reported outcome do you use in your patients with HF?

KCCQ

MLHFQ

EQ5D

Other (please specify):

None

12. Where do you think that patients with HF should be followed-up?

Always in secondary/tertiary care

In secondary/tertiary care only if there is worsening of clinical status

In secondary/tertiary care only if they have HFrEF

Other (please specify):

13. How often do you plan a follow-up visit for stable patients with HF with left ventricular ejection fraction ≤40% (i.e. HFrEF)?

Every 3 months or less

Every 6 months

Every 1 year

Every 2 years

Other (please specify):

14. How often do you plan a follow-up visit for stable patients with HF with left ventricular ejection fraction =40-50% (HFmrEF)?

Every 3 months

Every 6 months

Every 1 year

Every 2 years

Other (please specify):

15. How often do you plan a follow-up visit for stable patients with HF with left ventricular ejection fraction ≥50% (HFpEF)?

Every 3 months

Every 6 months

Every 1 year

Every 2 years

Other (please specify):

16. When do you refer your patients with HF to the HF nurse-led clinic?

On a regular basis

Only when up-titration of medications and optimization of the therapy is needed

Only when worsening of clinical status is observed

Never

Nurse-led clinic not available at my institution

17. How long after introduction of a new drug do you preferably see (in clinic) a patient with HFrEF?

1 week

2 weeks

1 months

Other, specify:

18. In your experience does the regular assessment in outpatient nurse clinic increase the adherence to evidence-based medical treatment of HFrEF compared to standard periodical evaluation by physicians?

No

Only for the elderly

Yes

I don’t know

**Section 3.** **Device treatments in patients with heart failure**

19. I would not recommend the implantation of an ICD for primary prevention of sudden cardiac death in a patient with an indication and a good clinical status if:

Age>70

Age>75

Age>80

I would recommend implantation regardless of age

20. I would recommend the implantation of an ICD for primary prevention of sudden cardiac death in a patient

Only if etiology is ischemic

Only if there is fibrosis at the cardiac magnetic resonance regardless of etiology

Regardless of etiology and fibrosis

21. How long after the institution of optimal pharmacological therapy do you wait to evaluate indication for ICD/CRT?

1 month

3 months

1 year

2 years

**Section 4. Pharmacological treatments in patients with heart failure**

22. Below which value of EF ACE inhibitors, angiotensin receptor blockers, mineralocorticoid receptor antagonists and sacubitril/valsartan been demonstrated to improve mortality/morbidity in heart failure?

60%

50%

40%

30%

23. Current evidence supports sacubitril/valsartan use in patients with heart failure with EF

≤40%

≤50%

≤60%

Regardless of EF

24. Why in your opinion should medical therapy for HFrEF be implemented as much as possible?

It is not necessary in stable patients

Because it reduces mortality/morbidity

To improve symptoms

Other (please specify):

25. Which is in your clinical practice the major obstacle to the starting of/dose titration of ACE inhibitors/angiotensin receptor antagonists/ sacubitril/valsartan?

Worsening renal function

Hypotension

Cough

Hyperkalemia

None

26. Which is in your clinical practice the major obstacle to the dose titration of mineralocorticoid receptor antagonists?

Worsening renal function

Hypotension

Hyperkalemia

None

27. In a patient with EF = 30%, NYHA class = III, eGFR = 45 ml/min/1.73m2, potassium = 4.7 mEq/L, would you initiate therapy with spironolactone?

Yes

No

Yes and I would check potassium/renal function after 1-2 weeks

28. In a patient with EF = 30%, NYHA class = III, eGFR = 45 ml/min/1.73m2, potassium = 5.7 mEq/L, receiving spironolactone

I stop spironolactone

I start a potassium but I keep spironolactone

I stop spironolactone and use SPS (e.g. resonium)

29. Which is in your clinical practice the major obstacle to the dose titration of sacubitril/ valsartan?

Worsening renal function

Hypotension

Hyperkalemia

None

30. According to current guidelines recommendation for HFrEF

Evidence based medical therapy should be introduced regardless of age

Evidence based medical therapy demonstrated efficacy only in the younger (<65 years old)

Evidence based medical therapy should be used at lower doses in the elderly

Evidence based medical therapy is not indicated in the elderly (> 80 years old)

31. In your practice how often do your patients with HFrEF achieve the 100% target dose of HF medications?

<25%

26-50%

51-75%

>75%

32. Based on your knowledge, which is the best strategy to maximize the adherence to guidelines for the management of HFrEF?

Therapeutic implementation during hospitalization

Systematic follow-up in tertiary care centers

Exclusive assessment by general practitioners

Regular evaluation by nurse-clinic

33. You do not initiate/uptitrate HFrEF medications if:

sBP<110 mmHg

sBP<100 mmHg

sBP<95 mmHg

sBP<90 mmHg

Other (please specify):

34. You do not initiate/uptitrate HFrEF medications if:

eGFR<60 ml/min/1.73m2

eGFR<45 ml/min/1.73m2

eGFR<30 ml/min/1.73m2

Other (please specify):

35. Trial evidence suggests that dapagliflozin

Improves renal outcomes

Worsen renal outcomes

Should not be used in patients with eGFR<45 ml/min/1.73m2

Should not be used in patients with eGFR<60 ml/min/1.73m2

36. I use SGLT2i in HFrEF

Only if there is concomitant diabetes

Always

They are not available

I do not have enough experience with this treatment

37. In your clinical practice what is the major limitation to the implementation of SGLT2i use in HFrEF:

Hypotension

Genital infections

Worsening renal function

Hypoglycemia

I am not concerned about these potential adverse events

Other (please specify):

38. In your experience, what is the MAIN reason patients with HFrEF do not receive optimal HF medical therapy

Patients or clinicians are concerned about side effects

Clinicians think that new drugs are too expensive or not cost effective

Clinicians think that their patient is different from those in the trials

Clinicians are not aware of all beneficial drugs and their indications

39. Have you ever used potassium-binders in your clinical practice in HF patients?

Yes

No

40. In a patient with HF, I do not use potassium-binders because

They are too expensive

There is not enough evidence

They are not available

Other treatments, e.g. SPS, are as effective and safe as potassium-binders

I use potassium binders for hyperkalemia

41. How often do you screen for iron deficiency in HFrEF patients?

At every outpatient visit

Every 6 month or less

Every year

Only if the patient reports changes in symptoms

Only if there is anemia

Never

42. In which proportion of HFrEF patients do you follow a conventional sequencing approach when introducing/up-titrating drugs (as opposed to a more personalized approach)?

0-25%

26-50%

51-75%

76-100%

**Thank you for your contribution!!!**

**Appendix 2. Answers to remaining survey questions.**

**Section 1. Background**

Participants’ age (Q1).

**Section 2. Management of heart failure patients**

How many HF patients do participants visit each week? (Overall and divided by specialty) (Q6).

Which patient reported outcome do you use in your patients with HF? (Q11)

Where do you think that patients with HF should be followed-up? (Q12)

When do you refer your patients with HF to the HF nurse-led clinic? (Q16)

How long after introduction of a new drug do you preferably see (in clinic) a patient with HFrEF? (Q17)

In your experience does the regular assessment in outpatient nurse clinic increase the adherence to evidence-based medical treatment of HFrEF compared to standard periodical evaluation by physicians? (Q18)

**Section 3. Device treatments in patients with heart failure**

I would not recommend the implantation of an ICD for primary prevention of sudden cardiac death in a patient with an indication and a good clinical status if age: (Q19)

I would recommend the implantation of an ICD for primary prevention of sudden cardiac death in a patient (Q20):

Q20 according to specialty:

How long after the institution of optimal pharmacological therapy do you wait to evaluate indication for ICD/CRT? (Q21)

**Section 4. Pharmacological treatments in patients with heart failure**

Why in your opinion should medical therapy for HFrEF be implemented as much as possible? (Q24)

In a patient with EF = 30%, NYHA class = III, eGFR = 45 ml/min/1.73m2, potassium = 4.7 mEq/L, would you initiate therapy with spironolactone? (Q27)

In a patient with EF = 30%, NYHA class = III, eGFR = 45 ml/min/1.73m2, potassium = 5.7 mEq/L, receiving spironolactone (Q28):

According to current guidelines recommendation for HFrEF (Q30):

In your practice how often do your patients with HFrEF achieve the 100% target dose of HF medications? (Q31)

Based on your knowledge, which is the best strategy to maximize the adherence to guidelines for the management of HFrEF? (Q32)

I use SGLT2i in HFrEF (Q36):

The main reason of GDMT underuse in HFrEF is (Q38):

Have you ever used potassium binders (Q39)?

In a patient with HF, I do not use potassium-binders because (Q40):

How often do you screen for iron deficiency in HFrEF patients? (Q41)
